# Supplementary material for: Personalized Management of Malignant and Non-Malignant Ectopic Mediastinal Thyroid: A Proposed 10-Item Algorithm Approach
Source: Cancers (Basel). 2024 May 14;16(10):1868. doi: 10.3390/cancers16101868 (PMC11120123; doi:10.3390/cancers16101868)
Supplement: Supplementary file 1 [file cancers-16-01868-s001.zip › cancers-2975667-supplementary.pdf]

Supplementary Materials

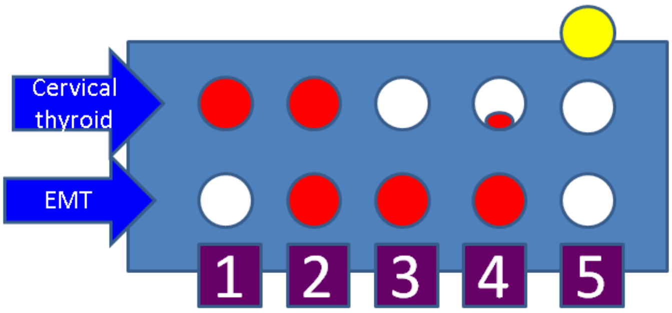

Figure S1. Qualitative perspective of the malignancies in EMT patients (red = cancer originating from the follicular thyroid cell at any differentiation level; white = benign thyroid tissue; yellow = non-thyroid type of cancer): 1 = subjects with primary cancer within the eutopic thyroid and benign EMT [22,35,123]; 2 = subjects with thyroid cancer in EMT and cervical thyroid (distinct foci) [36,42,55]; 3 = cancer in EMT, not in cervical thyroid [37,41,52,55,58,60–62,64,66,69–72]; 4 = malignancy in EMT with metastasis in cervical thyroid [55]; 5 = prior or concurrent non-thyroid malignancies at the moment of EMT identification in terms of originating from lung [11,49,101], breast [29,92,125] or ovary [89].
